# Developmental risk among Aboriginal children living in urban areas in Australia: the Study of Environment on Aboriginal Resilience and Child Health (SEARCH)

**DOI:** 10.1186/s12887-019-1902-z

**Published:** 2020-01-13

**Authors:** Shingisai Chando, Jonathan C. Craig, Leonie Burgess, Simone Sherriff, Alison Purcell, Hasantha Gunasekera, Sandra Banks, Natalie Smith, Emily Banks, Sue Woolfenden

**Affiliations:** 1 University of Sydney, Sydney, Australia; 2 Flinders University, Adelaide, Australia; 3 Sax Institute, Sydney, Australia; 4 Australian National University, Canberra, Australia; 5 Sydney Children’s Hospitals Network, Sydney, Australia; 6 Tharawal Aboriginal Medical Service, Campbelltown, Australia; 7 Riverina Medical and Dental Corporation, Wagga Wagga, Australia; 8 University of New South Wales, School of Women and Children’s Health, Sydney, Australia

**Keywords:** Child development, Pediatrics, Australian aboriginal, Caregiver concerns, Parents' Evaluation of Developmental Status

## Abstract

**Background:**

Most Australian Aboriginal children are on track with their development, however, the prevalence of children at risk of or with a developmental or behavioural problem is higher than in other children. Aboriginal child development data mostly comes from remote communities, whereas most Aboriginal children live in urban settings. We quantified the proportion of participating children at moderate and high developmental risk as identified by caregivers’ concerns, and determined the factors associated with developmental risk among urban Aboriginal communities.

**Methods:**

Study methods were co-designed and implemented with four participating urban Aboriginal Community Controlled Health Services in New South Wales, Australia, between 2008 and 2012. Caregiver-reported data on children < 8 years old enrolled in a longitudinal cohort study (Study of Environment on Aboriginal Resilience and Child Health: SEARCH) were collected by interview. The Parents’ Evaluation of Developmental Status (PEDS) was used to assess developmental risk through report of caregiver concerns. Odds ratios (OR) were calculated using multinomial logistic regression to investigate risk factors and develop a risk prediction model.

**Results:**

Of 725 children in SEARCH with PEDS data (69% of eligible), 405 (56%) were male, and 336 (46%) were aged between 4.5 and 8 years. Using PEDS, 32% were at high, 28% moderate, and 40% low/no developmental risk. Compared with low/no risk, factors associated with high developmental risk in a mutually-adjusted model, with additional adjustment for study site, were male sex (OR 2.42, 95% confidence intervals 1.62–3.61), being older (4.5 to < 8 years versus < 3 years old, 3.80, 2.21–6.54), prior history of ear infection (1.95, 1.21–3.15), having lived in 4 or more houses versus one house (4.13, 2.04–8.35), foster care versus living with a parent (5.45, 2.32–12.78), and having a caregiver with psychological distress (2.40, 1.37–4.20).

**Conclusion:**

In SEARCH, 40% of urban Aboriginal children younger than 8 years were at no or low developmental risk. Several factors associated with higher developmental risk were modifiable. Aboriginal community-driven programs to improve detection of developmental problems and facilitate early intervention are needed.

## Background

Although many Australian children do not have developmental problems, 20% of Australian children will start school without the developmental skills that they need to flourish at school [1–3]. For Aboriginal and Torres Strait Islander Australians (respectfully referred to as Aboriginal thereafter), this figure is estimated to be as high as 40% on the Australian Early Development Census, a population measure of school readiness [4]. Understanding factors related to this risk and identification of those children most at risk may accelerate timely universal and targeted interventions and lead to improved outcomes in school readiness, circumventing long-term adverse health, education and wellbeing outcomes [5–7]. A key challenge has been an inability to identify high risk groups in Australia, [5–7] such that around 20% of children with significant developmental problems are not identified before they start school [8].

Reducing disparities in early childhood development has been hampered by the lack of robust, population-based evidence to inform program development and implementation [5, 6]. While it is known that Aboriginal children are exposed to physical, family and social factors that increase their risk of developmental problems, such as ear infections, mental health problems in some caregivers, and inadequate housing, much of the current research is based either on small samples, in rural and remote settings and/or clinical settings [9–13]. Despite emerging data on the identification of Aboriginal children who are at developmental risk living in remote areas of Australia [14–16] the majority of Aboriginal children live in non-remote settings [17] and data on children from urban areas are particularly scant [18]. In Australia, research on early child developmental outcomes for urban Aboriginal children has been conducted only in a small birth cohort in south west Sydney of 114 children, the Gudaga study, which found that child development at 4.5 years was significantly below the standardised mean on a formal developmental assessment with strengths in the locomotor and personal-social skills [19]. Data on the broader population of urban Aboriginal children are lacking. These data are needed to better quantify how many urban Aboriginal children are at high risk of developmental problems, and inform assessments of service needs and interventions, including early identification [8, 18, 20].

The Study of Environment on Aboriginal Resilience and Child Health (SEARCH) is informed by a bioecological conceptual framework [21, 22] and is the largest longitudinal cohort study of urban Aboriginal children to date. The Study is focused on several community-identified health priorities, including healthy development, ear health, social and emotional wellbeing, children being placed into out of home care and housing. As SEARCH is based in New South Wales (NSW), the preferred term, Aboriginal, is used [23]. In this study of the subset of children aged 0 to less than 8 years in SEARCH, our aim is to quantify the proportion of participating children at moderate and high developmental risk as identified by caregivers’ concerns, and determine the factors associated with developmental risk among urban Aboriginal communities.

## Methods

### Design and sample

SEARCH collected baseline data between 2008 and 2012 [21]. The recruitment and selection methods from four participating Aboriginal Community Controlled Health Services (ACCHSs) are provided in the published study protocol [21]. In short, urban Aboriginal children aged 0 to 17 years and their caregivers who attended these ACCHSs were invited to participate by local Aboriginal research officers. The data presented here is the baseline data from 725 children aged less than 8 years enrolled through the four ACCHSs in NSW in the SEARCH cohort.

### Measures

Caregivers completed a baseline survey for themselves and for each of their children including demographic, physical, family and social data on variables relating to child health development and wellbeing. These questions focussed on the child, caregiver and environment level, in keeping with the underlying bioecological model underpinning SEARCH [22] and the community identified concerns regarding potential risk factors.

Factors assessed for this study are shown in Table 1. Child-level demographic and physical factors included sex, age, exposure to in-utero substances, being breastfed and ear infections. Age was categorised to reflect key transition points in a child’s early life - the first 1000 days (0 to < 3 years), the preschool years (3 to < 4.5 years) and early school years (4.5 to < 8 years).

**Table 1:** Characteristics of participants according to PEDS risk levels (*n* = 725)

	PEDS Risk Level				
	Low/No	Moderate	High	Total*	*p*-value^a^
	N (%)	N (%)	N (%)	N (%)	
CHILD FACTORS					
Sex					< 0.001
Female	155 (48)	84 (26)	81 (25)	320 (44)	
Male	138 (34)	119 (29)	148 (37)	405 (56)	
Age group (years)					< 0.001
< 3	108 (56)	54 (28)	31 (16)	193 (27)	
3 to < 4.5	70 (36)	71 (36)	55 (28)	196 (27)	
4.5 to < 8	115 (34)	78 (23)	143 (43)	336 (46)	
In utero cigarettes					0.2
No	135 (44)	91 (30)	79 (26)	305 (42)	
Yes	128 (39)	86 (26)	112 (34)	326 (45)	
In utero marijuana					0.1
No	217 (43)	147 (29)	139 (28)	503 (69)	
Yes	40 (36)	27 (24)	44 (40)	111 (15)	
Ear infection					0.002
No	145 (47)	89 (29)	72 (24)	306 (42)	
Yes	91 (34)	79 (29)	99 (37)	269 (37)	
Ever breastfed					0.8
No	112 (41)	77 (28)	85 (31)	274 (38)	
Yes	147 (43)	93 (27)	100 (29)	340 (47)	
FAMILY FACTORS					
Caregiver relationship to child					0.003
Parent	263 (43)	170 (28)	174 (29)	607 (84)	
Other relative	20 (34)	13 (22)	25 (43)	58 (8)	
Foster carer	9 (16)	18 (32)	29 (52)	56 (8)	
Parent/carer education					0.4
University	10 (23)	19 (44)	14 (33)	43 (6)	
Trade/certificate/diploma	106 (42)	65 (26)	79 (32)	250 (34)	
Year 11–12	36 (39)	22 (24)	34 (37)	92 (13)	
Year 10	59 (42)	37 (26)	46 (32)	142 (20)	
< Year 10	56 (44)	35 (28)	35 (28)	126 (17)	
Fortnightly household income (Australian $)					0.6
≥$2000	35 (47)	21 (28)	18 (24)	74 (10)	
$800 - $1999	91 (36)	72 (28)	93 (36)	256 (35)	
$600 - $799	45 (41)	31 (28)	33 (30)	109 (15)	
≤ $599	74 (43)	41 (24)	56 (33)	171 (24)	
Employment status					0.9
Employed (full or part-time)	69 (42)	47 (28)	50 (30)	166 (23)	
Unemployed/home duties/retired	203 (40)	137 (27)	164 (33)	504 (70)	
Government financial support^b^					0.3
None	33 (49)	18 (27)	16 (24)	67 (9)	
Family/parent/age only	227 (42)	147 (27)	172 (32)	546 (75)	
Disability/sickness/unemployment	19 (29)	20 (30)	27 (41)	66 (9)	
Carer allowance^c^					< 0.001
No	259 (43)	168 (28)	172 (29)	599 (83)	
Yes	20 (25)	17 (21)	43 (54)	80 (11)	
Parent/carer psychological distress (K10 score ≥ 22)					0.048
No	224 (43)	136 (26)	157 (30)	517 (71)	
Yes	37 (31)	32 (27)	51 (43)	120 (17)	
Parent/carer removal from family					0.1
No	254 (42)	167 (28)	183 (30)	604 (83)	
Yes	8 (22)	11 (31)	17 (47)	36 (5)	
SOCIAL FACTORS					
Housing					0.9
Own/mortgage	47 (42)	26 (23)	39 (35)	112 (15)	
Rent	54 (39)	37 (27)	48 (35)	139 (19)	
Public housing	168 (41)	114 (28)	124 (31)	406 (56)	
People/bedrooms					0.2
≤1	64 (43)	43 (29)	41 (28)	148 (20)	
1–2	129 (37)	102 (29)	117 (34)	348 (48)	
≥2	74 (47)	32 (20)	53 (33)	159 (22)	
Number of housing problem domains reported					0.1
0	103 (47)	55 (25)	63 (29)	221 (30)	
1	49 (36)	48 (36)	38 (28)	135 (19)	
2	43 (38)	24 (21)	45 (40)	112 (15)	
3+	82 (40)	57 (28)	68 (33)	207 (29)	
Number of houses lived in					< 0.001
1	93 (53)	53 (30)	29 (17)	175 (24)	
2	65 (44)	42 (28)	42 (28)	149 (21)	
3	31 (31)	32 (32)	37 (37)	100 (14)	
4+	25 (23)	28 (25)	58 (52)	111 (15)	

*Not all totals sum to 100% due to missing data. ^a^*P*-value is for the overall effect of the individual risk factor in the univariable multinomial nominal logistic regression, using imputed data. ^b^Government financial support provided to individuals and families in the form of payments or tax concessions. ^c^Government financial support provided to individuals providing care to another individual due to illness or disability

Family level factors were caregiver status, education, income, employment, receiving Carer’s Allowance (a government benefit for children with chronic health conditions e.g. autism, intellectual disability), mental health and experience of removal from the family as a child. Caregiver removal from their family, referred to the forced removal of the caregiver (when the caregiver was a child) from the natural family by a mission, the government or welfare. Income cut-offs were determined in keeping with the poverty lines for households with a single income earner versus couples in Australia. Caregiver psychological distress was defined as a Kessler-10 scale score of 22 or more [24, 25]. The Kessler-10 is a 10 item questionnaire designed to yield a global measure of distress and has been validated among Aboriginal adults in Australia [26].

The social level factors assessed were overcrowded housing, housing quality and residential mobility. The number of people per bedroom was calculated as the number of people living in the house divided by the number of bedrooms then categorised as ≤1(if more bedrooms than people), > 1 and < 2, ≥ 2. Housing quality was assessed using housing problem domains which were categorised as 0, 1, 2, ≥ 3 and included major electrical problems, major plumbing problems, damp or mildew on walls or ceilings or windows, no smoke alarm, house not secure, structural problems, and vermin. Residential mobility was examined using the number of houses lived in (1, 2, 3, ≥4) adapted from other published research in SEARCH [27]. Rules were used to set time-varying data (history of ear infection, number of houses lived in since birth) on the child survey to missing if specific conditions were met (see Additional file 2: Appendix 1 for rules).

### Parent’s evaluation of developmental status (PEDS)

All children enrolled in SEARCH who were less than 8-years old were eligible to be assessed for developmental risk using the Parents Evaluation of Development Status (PEDS) [28]. The PEDS is a screening tool that elicits caregiver concerns to quantify their child’s level of developmental risk and is widely used in population surveys and by healthcare providers internationally [20, 28, 29]. The PEDS has been used in populations varying in socio-economic status and cultures, including in Aboriginal children, and in community and clinical settings [3, 20, 30, 31]. The PEDS has a sensitivity of 91–97% and specificity of 73–86% in detecting children at high and/or moderate developmental risk in US studies [20]. The PEDS was administered by non-Aboriginal speech pathologists at the beginning of a speech and language assessment. The speech pathologists were trained by a PEDS trainer and some had experience working in Aboriginal communities prior to working in SEARCH. The PEDS is a 10 item questionnaire with open ended questions to elicit caregiver concerns about their child’s development, including behaviour. Concerns are covered by 10 domains: global/cognitive; expressive language and articulation; receptive language; fine motor; gross motor; behaviour; social and emotional; self-help; school; and other [30, 32]. Upon completion, the identified concerns are scored and rated as predictive, or non- predictive to determine the child’s risk level. The risk level is categorised as follows: low/no developmental risk (the reference category) = no predictive concerns; moderate developmental risk = 1 predictive concern; high developmental risk = 2 or more predictive concerns. Each of these categories has a specific service response. Caregiver concerns indicating that a child is at high developmental risk require a comprehensive developmental assessment and referrals to allied health therapy. Concerns indicating that a child has moderate developmental risk require a secondary screen with another developmental screening tool and low/no developmental risk children require parental education and ongoing monitoring.

### Statistical analysis

Due to the amount of missing data we used multiple imputation to account for the missing values for the risk factors. Under the assumption that the missing data were missing at random, multilevel multiple imputation was performed using REALCOM-IMPUTE software [33]. We created 50 imputed data sets, which incorporated variability due to uncertainty in the exact values, with a burn-in period of 2500 iterations and 500 iterations between imputations. Estimates of coefficients obtained for each dataset were combined using Rubin’s rules [34].

We analysed the associations between the individual risk factors and the risk of developmental problems as indicated by the PEDS pathway (high, moderate and low/no risk) in multinomial nominal logistic regression models, both unadjusted and adjusted for ACCHS, sex and age group. We used nominal rather than ordinal regression models as the proportional odds or parallel regression assumption was violated for several of the risk factors. In all models fitted, robust standard errors were used to account for the clustering of children within families.

To examine the association between multiple risk factors and level of developmental risk a prediction model was developed. All risk factors with an overall *p*-value < 0.2 in the unadjusted model were included in the initial multivariable multinomial logistic regression model. Backwards elimination was performed removing the least significant risk factor at each step, where *p*-values were greater than 0.05 until the final multivariable prediction model was obtained. The apparent performance of the prediction model was evaluated in terms of the Polytomous Discrimination Index (PDI) [35] and calibration [36]. The prediction model was internally validated and adjusted for overfitting by applying uniform shrinkage factors to the regression coefficients [37]. Further details of the prediction model development and validation can be found in the Additional file 2.

All analyses were performed using Stata version 14.2 (StataCorp, College Station, TX, USA) [34] with the exception of R (version 3.1.3: R Foundation for Statistical Computing, Vienna, Austria) for the discrimination and calibration calculations [35, 36].

## Results

### Characteristics of participants (Table 1)

Overall, 1669 children were enrolled in SEARCH. Of those 1045 were eligible for the PEDS screening and 725 children (69%) had a PEDS assessment. Of the 725 who were assessed 405 (56%) were male, 336 (46%) were aged between 4.5 and 8 years, 607 (84%) lived with a parent and 439 (60%) lived in households with a fortnightly income greater than $599. Three hundred and six children (42%) had a history of ear infections and 340 (47%) had ever been breastfed. For the majority of children, 503 (69%) had not been exposed to marijuana in utero, and for 571 children (71%) their caregivers were not experiencing psychological distress at the time of assessment.

**Fig. 1:**
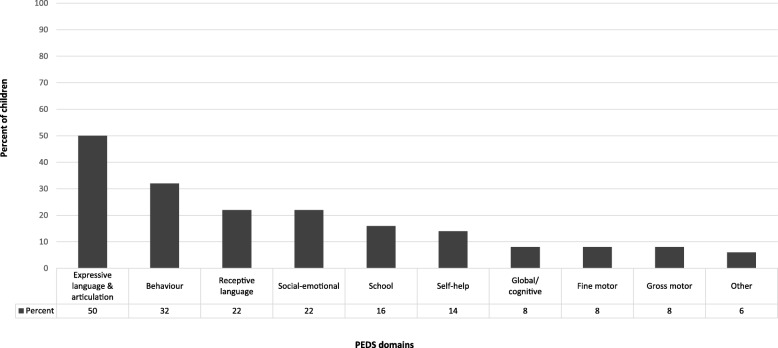
Prevalence of parental concerns by PEDS domain (*n* = 725)

Caregivers concerns on the PEDS indicated that 293 children (40%) were at no or low risk of developmental problems; 203 children (28%) were at moderate risk and 229 children (32%) were at high risk. The two most prevalent concerns were in the domains of expressive language and articulation, and behaviour (Fig. 1).

### Factors associated with moderate or high developmental risk (Table 2)

With adjustment for age group, sex and ACCHS, where appropriate, we found male gender, being 3 to < 4.5 years old versus < 3 years old and being in foster care versus living with a parent, were significantly associated with an increased likelihood of moderate or high developmental risk compared to low/no risk (Table 2). In utero marijuana exposure, a history of ear infections, having lived in 3 or more houses since birth versus one house, receiving the carers’ allowance and caregiver distress were also significantly associated with high developmental risk compared to low/no risk (Table 2).

**Table 2:** Multinomial logistic regression models, unadjusted and adjusted for ACCHS, age group and sex (*N* = 725)

	Unadjusted				Adjusted for ACCHS, age group and sex			
Risk factor	Moderate vs. low/no risk		High vs. low/no risk		Moderate vs. low/no risk		High vs. low/no risk	
	OR (95% CI)	*P* value	OR (95% CI)	*P* value	aOR (95% CI)	*P* value	aOR (95% CI)	*P* value
Sex								
Female (ref)	1	0.015	1	< 0.001	1	0.009	1	< 0.001
Male	1.59 (1.10, 2.31)		2.05 (1.43, 2.95)		1.65 (1.13, 2.41)		2.28 (1.56, 3.33)	
Age group								
< 3 years (ref)	1	0.014	1	< 0.001	1	0.006	1	< 0.001
3 to < 4.5 years	2.03 (1.26, 3.26)		2.74 (1.61, 4.65)		2.19 (1.35, 3.55)		3.04 (1.74, 5.31)	
4.5 to < 8 years	1.36 (0.89, 2.08)		4.33 (2.71, 6.93)		1.43 (0.93, 2.19)		4.41 (2.70, 7.19)	
In utero cigarettes								
No (ref)	1	0.917	1	0.13	1	0.726	1	0.069
Yes	1.02 (0.70, 1.49)		1.37 (0.91, 2.06)		1.07 (0.73, 1.59)		1.51 (0.97, 2.37)	
In utero marijuana								
No (ref)	1	0.999	1	0.092	1	0.788	1	0.029
Yes	1.00 (0.60, 1.66)		1.55 (0.93, 2.58)		1.07 (0.64, 1.81)		1.82 (1.06, 3.10)	
Ear infection								
No (ref)	1	0.113	1	0.003	1	0.170	1	0.005
Yes	1.39 (0.92, 2.10)		1.93 (1.25, 2.97)		1.35 (0.88, 2.06)		1.96 (1.23, 3.14)	
Ever breastfed								
Yes (ref)	1	0.510	1	0.522	1	0.719	1	0.720
No	1.14 (0.77, 1.68)		1.14 (0.76, 1.71)		1.08 (0.72, 1.62)		1.08 (0.70, 1.68)	
Number of houses lived in since birth								
1 (ref)	1	0.153	1	< 0.001	1	0.181	1	< 0.001
2	1.20 (0.72, 2.00)		1.86 (1.04, 3.30)		1.22 (0.73, 2.06)		1.78 (0.96, 3.30)	
3	1.68 (0.94, 3.00)		3.05 (1.66, 5.62)		1.68 (0.91, 3.07)		2.61 (1.35, 5.02)	
4+	1.81 (1.02, 3.21)		5.19 (2.78, 9.68)		1.82 (1.01, 3.31)		4.41 (2.25, 8.63)	
Relationship to child								
Parent (ref)	1	0.035	1	< 0.001	1	0.012	1	< 0.001
Other relative	1.05 (0.50, 2.22)		1.90 (0.87, 4.17)		0.94 (0.45, 2.00)		1.46 (0.60, 3.58)	
Foster carer	3.13 (1.32, 7.43)		4.84 (2.12, 11.10)		3.52 (1.53, 8.08)		5.10 (2.36, 11.02)	
Parent/carer education								
University (ref)	1	0.169	1	0.557	1	0.119	1	0.594
Trade/certificate/diploma	0.35 (0.15, 0.81)		0.55 (0.23, 1.33)		0.34 (0.15, 0.77)		0.54 (0.22, 1.34)	
Year 11–12	0.34 (0.14, 0.87)		0.68 (0.27, 1.73)		0.33 (0.13, 0.82)		0.75 (0.28, 1.98)	
Year 10	0.35 (0.15, 0.84)		0.57 (0.22, 1.44)		0.33 (0.14, 0.79)		0.61 (0.23, 1.61)	
< Year 10	0.36 (0.15, 0.89)		0.47 (0.18, 1.23)		0.36 (0.15, 0.87)		0.52 (0.19, 1.40)	
Fortnightly household income (Australian $)								
> = $2000 (ref)	1	0.597	1	0.257	1	0.682	1	0.347
$800 - $1999	1.31 (0.72, 2.40)		1.84 (0.97, 3.50)		1.29 (0.69, 2.42)		1.86 (0.94, 3.70)	
$600 - $799	1.15 (0.56, 2.34)		1.35 (0.63, 2.85)		1.10 (0.52, 2.34)		1.48 (0.65, 3.37)	
< = $599	0.97 (0.51, 1.86)		1.38 (0.71, 2.67)		0.98 (0.49, 1.93)		1.57 (0.75, 3.29)	
Employment status								
Employed (full or part-time) (ref)	1	0.993	1	0.639	1	0.947	1	0.571
Unemployed/Home duties/retired	1.00 (0.66, 1.52)		1.11 (0.71, 1.74)		0.99 (0.64, 1.52)		1.15 (0.71, 1.86)	
Housing								
Own/mortgage (ref)	1	0.794	1	0.734	1	0.813	1	0.808
Rent	1.20 (0.63, 2.28)		1.06 (0.56, 2.01)		1.20 (0.62, 2.32)		1.06 (0.53, 2.10)	
Public housing	1.18 (0.72, 1.93)		0.88 (0.52, 1.50)		1.18 (0.70, 1.98)		0.89 (0.51, 1.57)	
People per bedroom								
< =1 (ref)	1	0.112	1	0.373	1	0.099	1	0.297
> 1, < 2	1.13 (0.71, 1.80)		1.38 (0.85, 2.25)		1.10 (0.69, 1.76)		1.36 (0.79, 2.33)	
> =2	0.68 (0.39, 1.19)		1.12 (0.62, 2.01)		0.64 (0.36, 1.14)		0.95 (0.49, 1.83)	
Number of housing problem domains reported								
0 (ref)	1	0.180	1	0.339	1	0.248	1	0.429
1	1.75 (1.04, 2.93)		1.26 (0.73, 2.18)		1.71 (1.00, 2.93)		1.26 (0.68, 2.35)	
2	1.04 (0.56, 1.91)		1.68 (0.93, 3.00)		1.05 (0.56, 1.97)		1.66 (0.89, 3.07)	
3+	1.27 (0.80, 2.02)		1.36 (0.83, 2.22)		1.28 (0.79, 2.06)		1.32 (0.78, 2.25)	
Government financial support								
None (ref)	1	0.358	1	0.078	1	0.308	1	0.142
Family/Parent/Age only	1.16 (0.64, 2.08)		1.54 (0.81, 2.91)		1.21 (0.66, 2.21)		1.71 (0.83, 3.53)	
Disability/Sickness/Unemployment	1.81 (0.77, 4.25)		2.77 (1.14, 6.72)		1.91 (0.81, 4.51)		2.64 (1.00, 6.93)	
Carer allowance								
No (ref)	1	0.426	1	0.001	1	0.489	1	0.022
Yes	1.30 (0.68, 2.48)		3.15 (1.59, 6.23)		1.26 (0.66, 2.42)		2.44 (1.14, 5.24)	
Parent/carer psychological distress (K10 score > = 22)								
No (ref)	1	0.202	1	0.015	1	0.113	1	0.004
Yes	1.39 (0.84, 2.32)		1.85 (1.13, 3.04)		1.52 (0.91, 2.57)		2.23 (1.29, 3.86)	
Parent/carer removal from family								
No (ref)	1	0.133	1	0.032	1	0.104	1	0.057
Yes	2.00 (0.81, 4.92)		2.72 (1.04, 6.80)		2.13 (0.86, 5.31)		2.67 (0.97, 7.35)	

### Factors associated with moderate and/or high developmental risk: mutually adjusted model

In a mutually-adjusted model, the odds of caregiver concerns indicating high versus low/no developmental risk were significantly higher for children who were male, were at least 3 years old versus < 3 years old, had a history of ear infections, were in foster care versus living with a parent, had a caregiver reporting psychological distress and had lived in 3 or more houses since birth versus one house (Table 3). Caregiver concerns indicating moderate developmental risk were higher when compared to those indicating low/no developmental risk for children who were male, aged between 3 and 4.5 years versus < 3 years, and in foster care versus living with a parent (Table 3).

**Table 3:** Multivariable multinomial logistic regression: risk factors associated with moderate and high developmental risk on the PEDS (*n* = 725)

	Moderate vs. Low/No PEDS risk		High vs. Low/No PEDS risk	
	aOR (95% CI)	*p*-value	aOR (95% CI)	*p*-value^*^
Sex				
Female (ref)	1	0.007	1	< 0.001
Male	1.70 (1.15–2.50)		2.42 (1.62–3.61)	
Age group				
< 3 years (ref)	1	0.006	1	< 0.001
3 to < 4.5 years	2.24 (1.36–3.68)		3.06 (1.65–5.67)	
≥ 4.5 years	1.37 (0.88–2.13)		3.80 (2.21–6.54)	
Ever ear infection				
No (ref)	1	0.2	1	0.006
Yes	1.32 (0.86–2.03)		1.95 (1.21–3.15)	
Carer relationship with child				
Parent (ref)	1	0.012	1	< 0.001
Other relative	0.98 (0.46–2.07)		1.66 (0.70–3.91)	
Foster carer	3.61 (1.55–8.44)		5.45 (2.32–12.78)	
Psychological distress				
No (ref)	1	0.1	1	0.002
Yes	1.58 (0.93–2.67)		2.40 (1.37–4.20)	
Number of houses lived in				
1 (ref)	1	0.2	1	0.001
2	1.23 (0.73–2.07)		1.77 (0.95–3.29)	
3	1.62 (0.88–2.98)		2.49 (1.28–4.86)	
≥ 4	1.75 (0.95–3.23)		4.13 (2.04–8.35)	

*Analysis conducted using imputed data and adjusted for ACCHS, as well as the factors listed in this Table. *P*-value is significant when < 0.05

When the number of houses lived in since birth variable was included in the multivariable model as a continuous variable, the odds of caregiver concerns indicating moderate or high developmental risk versus low/no risk increased significantly for each additional house lived in (moderate versus low/no risk 1.22 (1.01, 1.47), high versus low/no risk 1.59 (1.28, 1.98).

From the prediction model, a child would be most likely to have a PEDS in the high developmental risk category if they had the following characteristics; were a boy aged between 4.5–8 years, were living in foster care, had a history of ear infections, had a caregiver in psychological distress and had more than four home moves since birth. Conversely, a child with the following characteristics would be least likely to have a PEDS in the high developmental risk category if they; were a girl aged less than 3 years, were living with their parents, had no history of ear infections, had a caregiver who did not report significant psychological distress and had only lived in one house (Additional file 1: Table S1).

## Discussion

Based on caregiver concerns on the PEDS, 40% of urban Aboriginal children aged less than 8 years in SEARCH were at no or low risk of developmental problems (no predictive concerns); for 60%, their caregivers had predictive concerns on the PEDS indicating moderate-high developmental risk. The prevalence of high developmental risk for urban Aboriginal children in this study (32%) was more than double the summary estimate of the global prevalence of concerns reported in a recent systematic review (13.8%) [20]. Of the 37 studies included in this review, the majority were among non-Indigenous populations in high-income countries, with eight conducted in low and middle-income countries and two in disadvantaged populations in the United States. Six of the studies included Indigenous populations in Australia [1] and the USA [5]. Our findings are consistent with the rate of developmental risk found in high risk US populations [38].

Using a developmental surveillance tool such as the PEDS gave Aboriginal caregivers a structured opportunity to express their concerns about their children’s development, in keeping with the values of SEARCH and its partner services. If developmental surveillance was undertaken in the ACCHSs using the PEDS, this research has highlighted the proportion of children at high and/or moderate developmental risk who would be identified early and require further assessment of their developmental concerns using the PEDS. It would also highlight high risk groups of children to support due to the presence of the associated risk factors. Of note, caregivers had the most concerns about expressive language and articulation, and behaviour. This is in keeping with research with other caregiver groups using the PEDS in Australia [3] and with data from SEARCH indicating elevated levels of speech and language issues and shortfalls in mainstream service provision [39]. This also highlights a caregiver need for services that support children’s speech and language and behaviour in ACCHs and mainstream services.

Previous research using the PEDS in a culturally and linguistically diverse and low socioeconomic status cohort of children in Australia showed that developmental risk increased with the age [33]. Similarly, in our cohort, children in the older age group (4.5 to 8 years) had the highest prevalence of developmental risk. This may be due to a greater awareness by caregivers of their child’s developmental status in relation to that of other children their age and involvement in school readiness programs. A US study found that parents generally derive their concerns and make judgments about their child’s development by comparing their child to others [29].

Middle ear conditions (e.g., acute otitis media and otitis media with effusion) may impact hearing, speech and language development, which may in turn adversely impact overall school readiness [40]. Among Aboriginal children, the prevalence of otitis media can range between 7 and 50% depending on whether the sample is urban or remote [41, 42] and findings from SEARCH indicate 29% of children aged 0–17 years had a current specialist-confirmed diagnosis of otitis media at baseline [21, 43]. We found an association between a history of ear infection and developmental risk, and access to quality ear health interventions could afford an opportunity to modify the developmental risk of Aboriginal children.

Family factors associated with an increased developmental risk have been identified in previous studies [20]. A child’s home environment can influence their growth and development. In our study, while the majority of caregivers were not reporting psychological distress at baseline, poor caregiver mental health was associated with concerns indicating a child is at high developmental risk. Mental health problems in caregivers are likely to be multifactorial and investigating the specific stressors experienced by caregivers was beyond the scope of this paper [20, 44, 45]. However, it should be noted that having a child with developmental issues may be a source of distress for some caregivers. These findings warrant further study regarding strategies to optimise the mental health of caregivers, to benefit not only the caregiver but the family as a whole.

Children in foster care were identified by their caregivers as particularly vulnerable to developmental risk. Urban Aboriginal children attending an out-of-home care clinic have been identified through a clinical audit to have significant barriers to accessing care, including inadequate integration of services and resourcing [12]. It is thus important to explore the potential of implementing multidisciplinary, culturally safe partnerships, and intensive and comprehensive supports for children in foster care and their caregivers to achieve optimal development [12, 46].

We demonstrated a linear relationship between the number of houses the child lived in since birth and developmental risk. This is in keeping with the results from a recent US study which showed an association between greater than two household moves in a year and delayed development [45]. It is unclear whether this is the result of a direct causal relationship between housing stability and development, or whether it relates to the factors underlying a lack of continuity in housing.

This examination of the baseline data from the SEARCH cohort is a cross-sectional study and so caution must be exercised in inferring causality for the relationships observed. We have plans to examine this in the planned trajectory studies of developmental risk in the follow up of the SEARCH cohort which is underway. Our prediction model has highlighted groups of urban Aboriginal children who are more likely to have caregiver concerns indicating high developmental risk. One previous smaller study demonstrated multiple factors associated with reduced developmental risk to be preschool attendance, and having 10 or more child-appropriate books in the home [19].

This is the largest study to date of caregiver concerns indicating developmental risk in urban Aboriginal children. However, its sample size limits reliable quantification of observed associations between the outcome and some of the less common exposures, as well as statistical interactions between the risk factors. We imputed the missing data so that we could use data from all the children with a PEDS assessment in the analysis. We also adjusted the prediction model for overfitting using the estimated shrinkage factors. It is also worth noting that the SEARCH cohort is not designed to be representative of the general population of urban Aboriginal children. It is the most up to date and complete data set on urban Australian children available in terms of their developmental risk and associated factors. Cohort studies provide valid and reliable data on associations and risk based on internal comparisons, including estimates of relative risk. Caution should be applied when generalising from absolute measures, such as prevalence [47, 48].

The PEDS screening tool has been used in urban Aboriginal communities in NSW, Australia as part of statewide developmental surveillance, in Victoria as part of a school entry survey and as a survey tool for Indigenous communities in the USA [20]. There is a service referral algorithm in these states for those children who are identified as being at moderate or high developmental risk requiring further referral and assessment. The PEDS does not however provide a clinical developmental assessment and thus the children who are identified by caregiver concerns as being at moderate and/or high developmental risk should not be assumed to have a developmental delay or disability. We are currently examining the relationship between level of developmental risk on the PEDS and identified speech and language and behavioural problems in the SEARCH cohort.

## Conclusion

Our findings indicate that working with caregivers of urban Aboriginal children and eliciting their concerns about their children’s development using a standardised developmental surveillance tool, the PEDS, can be useful for identifying children at moderate and high developmental risk. We have identified factors relating to this increased risk, including housing stability, ear health, caregiver mental health and being in foster care. These factors should be considered to support Aboriginal communities to develop policies and effective culturally appropriate programs and services. Better awareness of these risk factors and the usefulness of routine screening with tools such as the PEDS in developmental surveillance may assist clinicians in ACCHSs with early identification of children at moderate to high developmental risk and timely access to services. Further investigation of the longitudinal relationship of factors related to developmental outcomes over time, including in the SEARCH cohort, is required to provide evidence to support effective policies and programs to prevent urban Aboriginal children being at moderate and high developmental risk.

## Supplementary information

**Additional file 1: Table S1.** Predicted probabilities (%) of high developmental risk*.

**Additional file 2:** Additional statistical information.

## Data Availability

Data and material availability for the SEARCH study is subject to application approval by the SEARCH study executive and ACCHS CEO group.

## Abbreviations

ACCHS: Aboriginal Community Controlled Health Service
PEDS: Parents’ Evaluation of Developmental Status
SEARCH: Study of Environment on Aboriginal Resilience and Child Health
