# Supplementary material for: Developmental risk among Aboriginal children living in urban areas in Australia: the Study of Environment on Aboriginal Resilience and Child Health (SEARCH)
Source: BMC Pediatr. 2020 Jan 13;20:13. doi: 10.1186/s12887-019-1902-z (PMC6956483; doi:10.1186/s12887-019-1902-z)
Supplement: Supplementary file 1 — Additional file 1: Table S1. Predicted probabilities (%) of high developmental risk*. [file 12887_2019_1902_MOESM1_ESM.docx]

| **Table S1. Predicted probabilities (%) of high developmental risk*** | | | | | | | | | | | | | | | | | | |
| --- | --- | --- | --- | --- | --- | --- | --- | --- | --- | --- | --- | --- | --- | --- | --- | --- | --- | --- |
|  |  |  | Never had an ear infection diagnosed by a doctor | | | | | | | | Have had an ear infection diagnosed by a doctor | | | | | | | |
|  |  |  | Carer not in psych distress | | | | Carer in psych distress | | | | Carer not in psych distress | | | | Carer in psych distress | | | |
|  |  |  | No. of houses since birth | | | | No. of houses since birth | | | | No. of houses since birth | | | | No. of houses since birth | | | |
| **Sex** | **Age (yrs)** | **Carer-child relationship** | 1 | 2 | 3 | 4+ | 1 | 2 | 3 | 4+ | 1 | 2 | 3 | 4+ | 1 | 2 | 3 | 4+ |
|  | < 3 | Parent | 6 | 9 | 11 | 15 | 11 | 15 | 18 | 25 | 9 | 14 | 16 | 22 | 16 | 22 | 26 | 34 |
|  |  | Other relative | 9 | 13 | 16 | 21 | 15 | 21 | 25 | 33 | 14 | 19 | 23 | 30 | 22 | 30 | 35 | 44 |
|  |  | Foster carer | 16 | 22 | 25 | 33 | 25 | 32 | 36 | 45 | 23 | 30 | 34 | 43 | 33 | 42 | 46 | 55 |
| Female | 3 to <4.5 | Parent | 12 | 17 | 20 | 26 | 19 | 26 | 30 | 38 | 18 | 24 | 28 | 36 | 27 | 36 | 40 | 49 |
|  |  | Other relative | 17 | 23 | 27 | 35 | 27 | 35 | 39 | 48 | 24 | 32 | 37 | 46 | 36 | 46 | 50 | 59 |
|  |  | Foster carer | 26 | 33 | 37 | 46 | 37 | 45 | 49 | 58 | 34 | 43 | 47 | 56 | 46 | 55 | 59 | 67 |
|  | 4.5 to <8 | Parent | 15 | 21 | 25 | 33 | 25 | 33 | 37 | 47 | 22 | 30 | 35 | 44 | 34 | 44 | 48 | 58 |
|  |  | Other relative | 21 | 29 | 34 | 43 | 33 | 43 | 47 | 57 | 30 | 40 | 45 | 54 | 44 | 54 | 59 | 67 |
|  |  | Foster carer | 33 | 42 | 46 | 56 | 46 | 55 | 59 | 68 | 43 | 53 | 57 | 66 | 56 | 65 | 68 | 76 |
|  | < 3 | Parent | 11 | 15 | 18 | 24 | 18 | 24 | 28 | 36 | 16 | 22 | 26 | 34 | 25 | 33 | 38 | 47 |
|  |  | Other relative | 15 | 21 | 25 | 33 | 24 | 33 | 37 | 46 | 22 | 30 | 34 | 43 | 34 | 43 | 48 | 57 |
|  |  | Foster carer | 24 | 32 | 35 | 44 | 35 | 44 | 47 | 57 | 33 | 42 | 45 | 55 | 45 | 54 | 57 | 66 |
| Male | 3 to <4.5 | Parent | 19 | 26 | 29 | 38 | 29 | 37 | 41 | 51 | 27 | 35 | 39 | 48 | 39 | 48 | 52 | 61 |
|  |  | Other relative | 26 | 35 | 39 | 48 | 38 | 48 | 52 | 61 | 36 | 45 | 49 | 59 | 49 | 58 | 62 | 70 |
|  |  | Foster carer | 36 | 45 | 48 | 57 | 48 | 56 | 59 | 68 | 46 | 55 | 58 | 66 | 57 | 66 | 68 | 75 |
|  | 4.5 to <8 | Parent | 24 | 33 | 37 | 46 | 36 | 46 | 50 | 60 | 34 | 43 | 48 | 57 | 47 | 57 | 61 | 70 |
|  |  | Other relative | 33 | 42 | 47 | 57 | 46 | 56 | 61 | 69 | 44 | 54 | 58 | 67 | 58 | 67 | 70 | 78 |
|  |  | Foster carer | 45 | 55 | 58 | 67 | 58 | 66 | 69 | 77 | 56 | 65 | 68 | 75 | 67 | 75 | 77 | 83 |
| **Using the prediction model in Appendix 4. Green = low: ≤ 20%, yellow = moderate: 21%-40%, orange = high: 41%- 60%, red = very high: ≥ 61%. For example, a male child, aged greater than 4.5 years with a history of ear infection, whose carer was experiencing psychological distress, and had lived in more than 4 houses since birth had a predicted probability of 83% for parental concerns on the PEDS indicating high developmental risk. A boy of the same age, living with a parent and had only lived in one house since birth had a predicted probability of 24% and a girl of the same age, living with a parent and had only lived in one house since birth had a predicted probability of 15%.* | | | | | | | | | | | | | | | | | | |
