# Supplementary material for: Developmental risk among Aboriginal children living in urban areas in Australia: the Study of Environment on Aboriginal Resilience and Child Health (SEARCH)
Source: BMC Pediatr. 2020 Jan 13;20:13. doi: 10.1186/s12887-019-1902-z (PMC6956483; doi:10.1186/s12887-019-1902-z)
Supplement: Supplementary file 2 — Additional file 2: Additional statistical information. [file 12887_2019_1902_MOESM2_ESM.docx]

**Additional file 2**

**Appendix 1. Rules for time-varying data**

Time-varying data (ever had an ear infection, number houses lived in since birth) on the child survey were set to missing if:

- the PEDS was done more than 6 months before or after the child survey and at that time the child was less than 2 years old;
- the PEDS was done more than 1 year before or after the child survey and the child was 2 years old or more.

There was an exception if the PEDS assessment was done after the child survey and the response to the ‘ever ear infection’ was ‘yes’. In this case the response to ‘ever ear infection’ was left as ‘yes’ as the response would still have been ‘yes’ when the PEDS was done.

Sensitivity analyses were performed varying the time permitted between the child survey and the PEDS assessment and findings indicated that the choice of length of time permitted did not lead to different results.

**Appendix 2. Prediction model development and validation**

Using the imputed data, univariable multinomial nominal logistic regression identified 12 risk factors to include in the multivariable model (p < 0.2): sex, age group, in utero exposure to cigarette smoke, in utero exposure to marijuana, ever had an ear infection, number of houses the child has lived in since birth, relationship of carer to the child, people per bedroom, number of housing domain problems, carer in psychological distress, carer allowance, forced removal of carer from family. After backwards elimination, six risk factors remained in the final model: sex, age group, ever had an ear infection, number of houses the child has lived in since birth, relationship of carer to the child, carer in psychological distress. After reinserting all excluded variables into the model, none was statistically significant. We obtained the same risk factors in the final model if we included all potential risk factors in the initial multivariable model then performed backwards elimination. Tests for linear trend were performed using the number of houses lived in since birth risk factor as a continuous variable in the multivariable model. Since the outcome has three levels we obtained two equations from the model, one for moderate versus low/no PEDS risk and the other for high PEDS risk versus low/no PEDS risk. The third equation, for high vs moderate PEDS risk, can be derived from these two equations. Exponentiated coefficients represent conditional odds ratios or, equivalently, relative risk ratios.

We evaluated the apparent performance of the prediction model in terms of the Polytomous Discrimination Index (PDI)[^23^](#_ENREF_23) and calibration[^24^](#_ENREF_24). Discrimination represents the probability to correctly identify those with each outcome category. A value of 1/3 represents no discrimination and a value of 1 represents perfect discrimination. Calibration compares the predicted probabilities from the model with the observed values by using a multinomial logistic regression to model the outcome as a function of the linear predictors from the prediction model. Values of 0 and 1 for the calibration intercepts and slopes, respectively, reflect perfect calibration. Predicted risks are considered to be underestimated if the calibration intercepts are >0, and overestimated if the calibration intercepts are <0. Discrimination and calibration of the prediction model are referred to as the apparent performance of the model. The median and range of the model performance measures across the 50 imputations are recommended as robust summary measures for combining these estimates after multiple imputation[^42^](#_ENREF_42). The prediction model was then internally validated using 200 bootstrap samples, sampling with replacement. Within each bootstrap sample, we repeated the variable selection process to obtain a final bootstrap model. The optimism of the prediction model was calculated as the mean difference between the bootstrap performance (PDI of the bootstrap model in each bootstrap sample) and the test performance (PDI of the bootstrap model in the original sample). We adjusted for overfitting by applying uniform shrinkage factors (estimates of the calibration slopes) to the regression coefficients[^25^](#_ENREF_25).

For apparent performance, the overall discrimination of the prediction model was 54% (range across 50 imputations: 53%, 55%), which represents the chance that a child from a randomly selected category of PEDS risk in a set of three children (one from each PEDS risk category) is correctly identified. The category specific probabilities of correctly identifying a child as having low/no, moderate and high PEDS risk in a set of children are 57% (range: 55%, 58%), 46% (range: 44%, 47%) and 60% (range: 59%, 62%), respectively. The optimism was 4.7% overall, and 4.0%, 5.9% and 4.0% for low/no, moderate and high PEDS risk, respectively. After adjustment for optimism, the overall discrimination reduced to 50% (range: 48%, 51%), category specific discrimination for low/no, moderate and high PEDS risk reduced to 53% (range: 51%, 54%), 40% (range: 38%, 41%) and 56% (range: 54%, 58%), respectively.

The prediction model is well-calibrated in the original sample and this is expected since the calibration is performed on the same data used to develop the prediction model. The calibration intercepts are close to zero with the moderate PEDS risk vs low/no PEDS risk intercept of 0.001 (range: -0.018, 0.013) and high PEDS risk vs low/no PEDS intercept of -0.001 (range: -0.039, 0.035). The calibration slopes are close to 1 with estimates of 0.998 (range: 0.920, 1.086) and 0.994 (range: 0.920, 1.054) for moderate and high PEDS risk vs low/no PEDS risk, respectively. The internal validation procedure provided two shrinkage factors of 0.701 for moderate vs low/no PEDS risk coefficients, and 0.807 for high vs low/no PEDS risk coefficients to correct for overfitting in the prediction model. The coefficients were multiplied by the corresponding shrinkage factor and the intercepts were re-estimated to ensure that overall calibration was maintained. The mean of the coefficients for ACCHS was incorporated in the intercepts. Prediction model coefficients before and after shrinkage are shown in Table B with the PEDS prediction model coefficients before and after shrinkage.

The prediction model with an example is presented in Appendix 4. Predicted probabilities for parental concerns indicating high PEDS risk for all combinations of the 6 risk factors are shown in Table 3. However, the predicted probability of a child having parental concerns indicating each of the three levels of PEDS risk can be evaluated using the model.

| **Table A. PEDS Internal validation results** | | | | | | | | | | | |  |
| --- | --- | --- | --- | --- | --- | --- | --- | --- | --- | --- | --- | --- |
| **Discrimination** | **Polytomous discrimination index (range)** | | | | | | | | | | |  |
|  | **Overall** | | | **Low/no PEDS risk** | | **Moderate PEDS risk** | | | **High PEDS risk** | | |  |
|  |  | | |  | |  | | |  | | |  |
| Apparent performance | 0.541 (0.529,0.553) | | 0.565 (0.548,0.581) | | | 0.455 (0.441,0.471) | | 0.602 (0.586,0.620) | | | |  |
| Bootstrap performance | 0.554 (0.548,0.558) | | 0.567 (0.560,0.575) | | | 0.487 (0.482,0.492) | | 0.608 (0.599,0.616) | | | |  |
| Test performance | 0.508 (0.502,0.512) | | 0.527 (0.520,0.535) | | | 0.428 (0.422,0.433) | | 0.568 (0.559,0.576) | | | |  |
|  |  | |  | | |  | |  | | | |  |
| Optimism | 0.047 (0.046,0.048) | | 0.040 (0.039,0.041) | | | 0.059 (0.058,0.061) | | 0.040 (0.039,0.042) | | | |  |
| Optimism adjusted performance | 0.495 (0.483,0.506) | | 0.525 (0.507,0.541) | | | 0.396 (0.382,0.411) | | 0.563 (0.546,0.581) | | | |  |
|  |  | | |  | |  | | |  | | |  |
| **Calibration** | **Moderate vs Low/no PEDS risk** | | | | | **High vs Low/no PEDS risk** | | | | | |  |
|  | **Intercept** | | **Slope** | | | **Intercept** | | | | **Slope** | |  |
|  | **Median (range)** | | **Median (range)** | | | **Median (range)** | | | | **Median (range)** | |  |
|  |  | |  | | |  | | | |  | |  |
| Original sample | 0.001 (0.018,0.013) | | 0.998 (0.920,1.086) | | | -0.001 (0.039,0.035) | | 0.994 (0.920,1.054) | | | |  |
| Bootstrap sample | 0.001 (0.008,0.008) | | 0.994 (0.958,1.037) | | | 0.000 (0.021,0.017) | | 0.997 (0.959,1.019) | | | |  |
| Test sample | 0.007 (0.002,0.015) | | 0.701(0.667,0.738) | | | 0.008 (-.014,0.024) | | 0.800 (0.774,0.827) | | | |  |
| **Table B. PEDS prediction model coefficients before and after shrinkage** | | | | | | | | | | | | |
|  | | **Coefficients (no shrinkage)** | | | | | **Coefficients (after shrinkage)** | | | | | |
|  | | **Moderate vs Low** | | | **High vs Low** | | **Moderate vs Low** | | | | **High vs Low** | |
|  | | **PEDS risk** | | | **PEDS risk** | | **PEDS risk** | | | | **PEDS risk** | |
| **Shrinkage factor** | |  | | |  | | 0.700705 | | | | 0.806824 | |
| **Intercept** | | -1.344383 | | | -3.166209 | | -1.055110 | | | | -2.606897 | |
| **Sex** | |  | | |  | |  | | | |  | |
| Female (ref) | | 0 | | | 0 | | 0 | | | | 0 | |
| Male | | 0.530125 | | | 0.883458 | | 0.371461 | | | | 0.712795 | |
| **Age group** | |  | | |  | |  | | | |  | |
| < 3 years (ref) | | 0 | | | 0 | | 0 | | | | 0 | |
| 3 to < 4.5 years | | 0.806584 | | | 1.117675 | | 0.565177 | | | | 0.901767 | |
| >= 4.5 years | | 0.315502 | | | 1.336300 | | 0.221074 | | | | 1.078158 | |
| **Ever ear infection** | |  | | |  | |  | | | |  | |
| No (ref) | | 0 | | | 0 | | 0 | | | | 0 | |
| Yes | | 0.279331 | | | 0.666846 | | 0.195728 | | | | 0.538027 | |
| **Number of houses lived in** | |  | | |  | |  | | | |  | |
| 1 (ref) | | 0 | | | 0 | | 0 | | | | 0 | |
| 2 | | 0.206056 | | | 0.571809 | | 0.144384 | | | | 0.461349 | |
| 3 | | 0.481511 | | | 0.913514 | | 0.337397 | | | | 0.737045 | |
| >=4 | | 0.562441 | | | 1.417418 | | 0.394105 | | | | 1.143607 | |
| **Carer relationship with child** | | | | |  | |  | | | |  | |
| Parent (ref) | | 0 | | | 0 | | 0 | | | | 0 | |
| Other relative | | -0.022900 | | | 0.507023 | | -0.016046 | | | | 0.409078 | |
| Foster carer | | 1.284476 | | | 1.695608 | | 0.900038 | | | | 1.368057 | |
| **Psychological distress** | |  | | |  | |  | | | |  | |
| No (ref) | | 0 | | | 0 | | 0 | | | | 0 | |
| Yes | | 0.454289 | | | 0.874732 | | 0.318322 | | | | 0.705755 | |

**Appendix 4. Prediction model**

The linear predictor for moderate PEDS risk vs low/no PEDS risk:

LP_1_ = -1.055 + (0.371 if male) + (0.565 if aged 3 to <4.5 years) + (0.221 if aged 4.5 to <8 years) + (0.196 if ever had an ear infection) + (0.144 if houses lived in = 2) + (0.337 if houses lived in = 3) + (0.394 if houses lived in ≥ 4) + (-0.016 if carer is other relative) + (0.900 if carer is foster carer) + (0.318 if carer in psychological distress).

The linear predictor for high PEDS risk vs low/no PEDS risk:

LP_2_ = -2.607 + (0.713 if male) + (0.902 if aged 3 to <4.5 years) + (1.078 if aged 4.5 to <8 years) + (0.538 if ever had an ear infection) + (0.461 if houses lived in = 2) + (0.737 if houses lived in = 3) + (1.144 if houses lived in ≥ 4) + (0.409 if carer is other relative) + (1.368 if carer is foster carer) + (0.706 if carer in psychological distress).

Then the predicted probabilities are

P(No/low PEDS risk) = 1 /(1 + exp(LP_1_) + exp(LP_2_)),

P(Moderate PEDS risk) = exp(LP_1_) /(1 + exp(LP_1_) + exp(LP_2_)),

P(High PEDS risk) = exp(LP_2_) /(1 + exp(LP_1_) + exp(LP_2_)).

In order to illustrate how to use the prediction model we have provided an example.

If we have a 7 year old boy, who has had at least one ear infection diagnosed by a doctor, has lived in 5 houses since birth, has a carer who is a parent and not in psychological distress:

LP_1_ = -1.055 + 0.371 + 0.221 + 0.196 + 0.394 = 0.127

LP_2_ = -2.607 + 0.713 + 1.078 + 0.538 + 1.144 = 0.866

P(No/low PEDS risk) = 1 /(1 + exp(0.127) + exp(0.866)) = 0.221,

P(Moderate PEDS risk) = exp(0.127) /(1 + exp(0.127) + exp(0.866)) = 0.252,

P(High PEDS risk) = exp(0.866) /(1 + exp(0.127) + exp(0.866)) = 0.527.

Therefore the predicted probabilities that this child has low/no PEDS risk is 22%, moderate PEDS risk is 25%, and high PEDS risk is 53%.
